# Genome-wide analysis of cytochrome P450s of *Trichoderma* spp.: annotation and evolutionary relationships

**DOI:** 10.1186/s40694-018-0056-3

**Published:** 2018-06-04

**Authors:** Sonia Chadha, Sayaji T. Mehetre, Ravindra Bansal, Alan Kuo, Andrea Aerts, Igor V. Grigoriev, Irina S. Druzhinina, Prasun K. Mukherjee

**Affiliations:** 1 Nuclear Agriculture and Biotechnology Division, Bhabha Atomic Research Centre, Trombay, Mumbai 400085 India; 2 U.S. Department of Energy Joint Genome Institute, Walnut Creek, CA 94598 USA; 3 Research Area Biochemical Technology, Institute of Chemical and Biological Engineering, TU Wien, 1060 Vienna, Austria

## Abstract

**Background:**

Cytochrome P450s form an important group of enzymes involved in xenobiotics degradation and metabolism, both primary and secondary. These enzymes are also useful in industry as biotechnological tools for bioconversion and a few are reported to be involved in pathogenicity. *Trichoderma* spp. are widely used in industry and agriculture and are known for their biosynthetic potential of a large number of secondary metabolites. For realising the full biosynthetic potential of an organism, it is important to do a genome-wide annotation and cataloguing of these enzymes.

**Results:**

Here, we have studied the genomes of seven species (*T. asperellum*, *T. atroviride*, *T. citrinoviride*, *T. longibrachiatum*, *T. reesei *, *T. harzianum* and *T. virens*) and identified a total of 477 cytochrome P450s. We present here the classification, evolution and structure as well as predicted function of these proteins. This study would pave the way for functional characterization of these groups of enzymes and will also help in realization of their full economic potential.

**Conclusion:**

Our CYPome annotation and evolutionary studies of the seven *Trichoderma* species now provides opportunities for exploration of research-driven strategies to select *Trichoderma* species for various applications especially in relation to secondary metabolism and degradation of environmental pollutants.

**Electronic supplementary material:**

The online version of this article (10.1186/s40694-018-0056-3) contains supplementary material, which is available to authorized users.

## Background

*Trichoderma* (Hypocreales, Ascomycota, Dikarya) species are among the most common fungi frequently isolated as mycotrophs from various fungi and as saprotrophs from free soil, soil litter, dead wood and rhizosphere, and includes more than 256 accepted species [1, 2]. These fungi are economically important due to their ability to produce enzymes of industrial importance, ability to kill/inhibit many plant pathogenic fungi, to boost plant immunity and promote plant growth, in addition to their ability to produce a plethora of secondary metabolites [3, 4]. A few species/strains are known to be opportunistic human pathogens [5]. *Trichoderma* spp. are thus ideal candidates for genome-wide studies to further augment their biotechnological applications. The first species to be sequenced is *Trichoderma reesei*, industrial source of cellulases and hemicellulases [6]. This was soon followed by whole genome sequencing of two strongly mycoparasitic species, viz. *T*. *atroviride* and *T*. *virens* [7]. A comparative analysis of the mycoparasitic species i.e., *T*. *atroviride* and *T*. *virens* with that of weaker mycoparasitic species *T*. *reesei* yielded novel information on the genome-scale differences between these species. In general, the mycoparasitic species are enriched in genes involved mycoparasitism and secondary metabolism [1, 7, 8]. Four more species, i.e., *T*. *asperellum* and *T*. *harzianum* (biocontrol species) and *T*. *longibrachiatum* and *T*. *citrinoviride* (opportunistic human pathogens) were subsequently sequenced by US Department of Energy Joint Genome Initiative (Mycocosm [9]; http://jgi.doe.gov/fungi). However, detailed analyses of these four genomes are awaited.

Cytochrome P450 genes (CYPs) are found in the genomes of prokaryotes and lower and higher eukaryotes. CYPs constitute a large superfamily of heme-thiolate proteins involved in the metabolism of a wide variety of both exogenous and endogenous compounds [10]. CYPs are heme b containing monooxygenases which were recognized and defined as a distinct class of hemoproteins [11]. Cyp proteins catalyze the regio-, chemo- and stereospecific oxidation of a vast number of substrates under mild reaction conditions, thus accomplishing chemical transformations. These functions make them important players in xenobiotic degradation and in primary and secondary metabolism. A few such enzymes are also reported to be involved in pathogenicity of plant pathogenic fungi [12–16]. Their diverse functional properties reflect their biological roles and make them important candidates for extensive investigation to explore diverse aspects of P450 functions and regulation as well as for biotechnological applications [17, 18].

Cytochrome P450s are categorized into two main classes, B (initially assigned as Bacterial) and E (initially assigned as Eukaryotic). Bacterial P450s with three component systems [an FAD-containing flavoprotein (NADPH or NADH-dependent reductase), an iron sulphur protein, and the P450 hemeprotein] and the fungal P450 nor (nitric oxide reductase). Clan CYP 55 belong to the ‘B’-class [19]. All the other known P450s from distinct systems, including eukaryotic and bacterial P450s, belong to the ‘E’-class. The eukaryotic microsomal P450 system contains two components, the NADPH:P450 oxidoreductase (POR), a flavoprotein containing both FAD and FMN, and the P450 monooxygenase containing the heme domain. The prokaryotic (bacterial) soluble P450 monooxygenase P450BM3 (Cyp102) exists as a single protein with both heme and flavin functional domains.

The complete CYP complement of one organism, called CYPome, is a collection of CYP genes in the genome of that species [20]. The current state of knowledge on P450 evolution in eukaryotes points to CYP51 as the ancestral P450, which is believed to have led to the evolution of all the present day P450 families [21]. The expansion and diversification of CYPomes may also provide information on fungal evolutionary adaptation to ecological niches. A key development affecting applied P450 research is the need to define and annotate ever-expanding genomic information. Various web-based resources have been developed to probe and assign various orphan CYPs in numerous genomes, owing to the identification of conserved motifs responsible for oxygen and heme-binding. These databases reveal that enormous number of sequence-diverse P450s is yet to be discovered and explored for functions and diverse activities in all kingdoms. One of the most commonly used resources includes the Nelson database (http://drnelson.uthsc.edu/cytochromeP450.html) [21]. The grouping scheme for CYPs is based on amino acid sequence similarity [22]. The original nomenclature for CYPs is based upon amino acid identity where Cyp proteins with at least 40% identity are placed in the same family [22, 23]. However, due to various evolutionary mechanisms, a straight forward nomenclature might be difficult, therefore, family definition is recommended by integrating phylogeny and protein evolution [24]. To each family, Cyp number is designated according to their taxonomic groups. Fungal Cyp families are numbered as Cyp51-Cyp69, Cyp501-Cyp699 and Cyp5001-Cyp6999. With rapid increase in discoveries of new Cyp proteins through genome sequencing, Nelson database lacks efficiency to annotate all Cyp proteins. For higher-level grouping of families identified via the sequence similarity-based scheme, CYP clan system was first developed and then applied to classify metazoan CYPs [25]. The CYP clan approach places all Cyp families with a monophyletic origin into a single clan and has been successfully applied to classify Cyp families in fungi [26]. For example, if new Cyps had equal identity to two or more Cyp families, they can be tentatively assigned to a clan in which these families belong. A site dedicated to filamentous fungi has been developed that includes comprehensive information on P450 clans and families (http://p450.riceblast.snu.ac.kr) [27]. In filamentous fungi, CYPs are involved in various physiological processes including fitness, resistance to xenobiotics and biosynthesis of a vast array of secondary metabolites with applications in biomedical, agricultural and industrial fields [28–31].

Keeping in view the wide spectrum of biotechnological applications of *Trichoderma* species, and the important role that CYPs play in the biology of fungi, we decided to annotate and make an inventory of the CYPome in the seven species of *Trichoderma* that have been sequenced by JGI. Annotation of these genes would help in commercial exploitation of these proteins. Earlier, the CYPome of several fungal species have been analysed in detail, e.g., *Aspergillus nidulans* [29], *Phanerochete chrysosporium* [32], *Mycosphaerella graminicola* [33] and *Grosmannia clavigera* [34]. However, this subject has not been covered in earlier analyses of *Trichoderma* genomes, except for the inclusion of *T*. *reesei* in a broad analysis of fungal CYPomes [35]. Moreover, a detailed phylogenetic analysis of *Trichoderma* CYPome could advance our understanding of the evolutionary processes of cytochrome P450 proteins in fungi.

## Results

### CYP proteins in *Trichoderma*

*Trichoderma* CYPome embodies a group of cytochrome P450 diverse proteins which are predicted to participate in a spectrum of functions involved in primary, secondary and xenobiotic metabolism. A total of 595 cytochrome P450 proteins have been identified in seven *Trichoderma* species. These entries were further analysed for the presence of full cytochrome P450 domain which led to the selection of a total 477 Cyp proteins (Table 1) for the detailed study. Entries with incomplete sequences and domains are listed in Additional file 1: Table S1.

**Table 1:** Taxonomic distribution of putative CYPs in seven *Trichoderma* species

Species	Genome size (Mb)	No. of predicted genes	Total Cyp proteins	Proteins with complete sequences	Clan type	Family type	Families with no FCPD matches
*T. asperellum*	37.46	12,586	73	62	25	40	7
*T. atroviride*	36.10	11,863	69	57	22	36	5
*T. citrinoviride*	33.48	9397	75	57	23	41	6
*T. longibrachiatum*	32.24	10,792	68	53	21	38	4
*T. reesei*	34.10	9129	70	57	23	42	4
*T. harzianum*	40.98	14,095	118	101	31	67	12
*T. virens*	39.00	12,427	122	90	31	59	12

Analysis showed that *T. harzianum* genome harbours the highest number of Cyps (101), followed by *T. virens* (90), *T. asperellum* (62), *T. atroviride* (57), *T. citrinoviride* (57), *T. reesei* (57) *and T. longibrachiatum* (53). The number of Cyp proteins for families Cyp5080, Cyp52, Cyp534, Cyp535, Cyp541 and Cyp618 were found conserved among seven *Trichoderma* species. Cytochrome P450 families Cyp504, Cyp505, Cyp5080, Cyp51, Cyp52, Cyp528, Cyp534, Cyp535, Cyp539, Cyp541, Cyp548, Cyp570, Cyp58, Cyp584, Cyp61, Cyp618, Cyp620, Cyp65 and Cyp671 were found ubiquitously present in *Trichoderma* suggesting a conserved role of these proteins. In *Trichoderma*, Cyp families Cyp5039, Cyp5044, Cyp5046, Cyp5049, Cyp5055, Cyp5057, Cyp5060, Cyp5128, Cyp5129, Cyp5134, Cyp5168, Cyp5181, Cyp5246, Cyp5262, Cyp5268, Cyp5292, Cyp5296, Cyp5320, Cyp5334, Cyp5390 and Cyp5391 didn’t have any matches in Fungal Cytochrome P450 Database (FCPD). The cytochrome P450 families unique to *Trichoderma* were identified as Cyp5039, Cyp5049, Cyp5055, Cyp5057, Cyp5128, Cyp5129, Cyp5134, Cyp5268, Cyp5292, Cyp5296, Cyp5390 and Cyp5391. These families were predicted to be involved in both xenobiotic and secondary metabolism (Table 2).

**Table 2:** Phylogenetic clustering of *Trichoderma* CYP families and clans

Phylogenetic group ID	Total entries	CYP family	CYP Clan	Putative functions
1	33	Cyp5044^a^, Cyp5078, Cyp5080, Cyp5104, Cyp528, Cyp531, Cyp532, Cyp5320^a^, Cyp631	CYP528, CYP531, CYP532	Xenobiotic metabolism
2	19	Cyp535, Cyp570	CYP507	Xenobiotic metabolism
3	3	Cyp673	CYP673	
4	11	Cyp5055^a^, Cyp5057^a^, Cyp5262^a^, Cyp537, Cyp62, Cyp684	CYP537, CYP62	Xenobiotic metabolism Secondary metabolism
5	35	Cyp5039^a^, Cyp5094, Cyp5128^a^, Cyp5129^a^, Cyp5292^a^, Cyp551, Cyp552, Cyp58, Cyp677, Cyp680, Cyp682	CYP58, CYP677	Secondary metabolism Xenobiotic metabolism
6	10	Cyp5246^a^, Cyp53	CYP53	Xenobiotic metabolism
7	3	Cyp630	CYP630	Primary metabolism
8	23	Cyp574, Cyp5076, Cyp5168^a^, Cyp671	CYP574	Secondary metabolism
9	17	Cyp548	CYP548	Xenobiotic metabolism
10	56	Cyp5117, Cyp561, Cyp563, Cyp65	CYP65	Secondary metabolism
11	3	Cyp627	CYP627	
12	62	Cyp5049^a^, Cyp52, Cyp5296^a^, Cyp538, Cyp539, Cyp584, Cyp587, Cyp655	CYP52, CYP59	Xenobiotic metabolism
13	19	Cyp5181^a^, Cyp5334^a^, Cyp534, Cyp613, Cyp685	CYP534, CYP613	Xenobiotic metabolism
14	39	Cyp5134^a^, Cyp526, Cyp5390^a^, Cyp617, Cyp618	CYP526, CYP547	Secondary metabolism
15	36	Cyp505, Cyp5099, Cyp540, Cyp541	CYP505, CYP540, CYP56	Primary metabolism
16	9	Cyp504	CYP504	Xenobiotic metabolism
17	51	Cyp5046^a^, Cyp5068, Cyp5268^a^, Cyp530, Cyp5391^a^, Cyp620, Cyp621	CYP530, CYP533	Xenobiotic metabolism
18	1	Cyp5042	CYP5042	
19	18	Cyp503, Cyp5090, Cyp559, Cyp611, Cyp635, Cyp636, Cyp641, Cyp642	CYP54, CYP550, CYP559, CYP642, CYP657, CYP659	Secondary metabolism
20	29	Cyp5060^a^, Cyp51, Cyp55, Cyp61	CYP51, CYP55, CYP61	Primary metabolism

^a^Corresponding clans for these families are absent in FCPD

### Abundance and diversity of cytochrome P450 family/clan

**Fig. 1:**
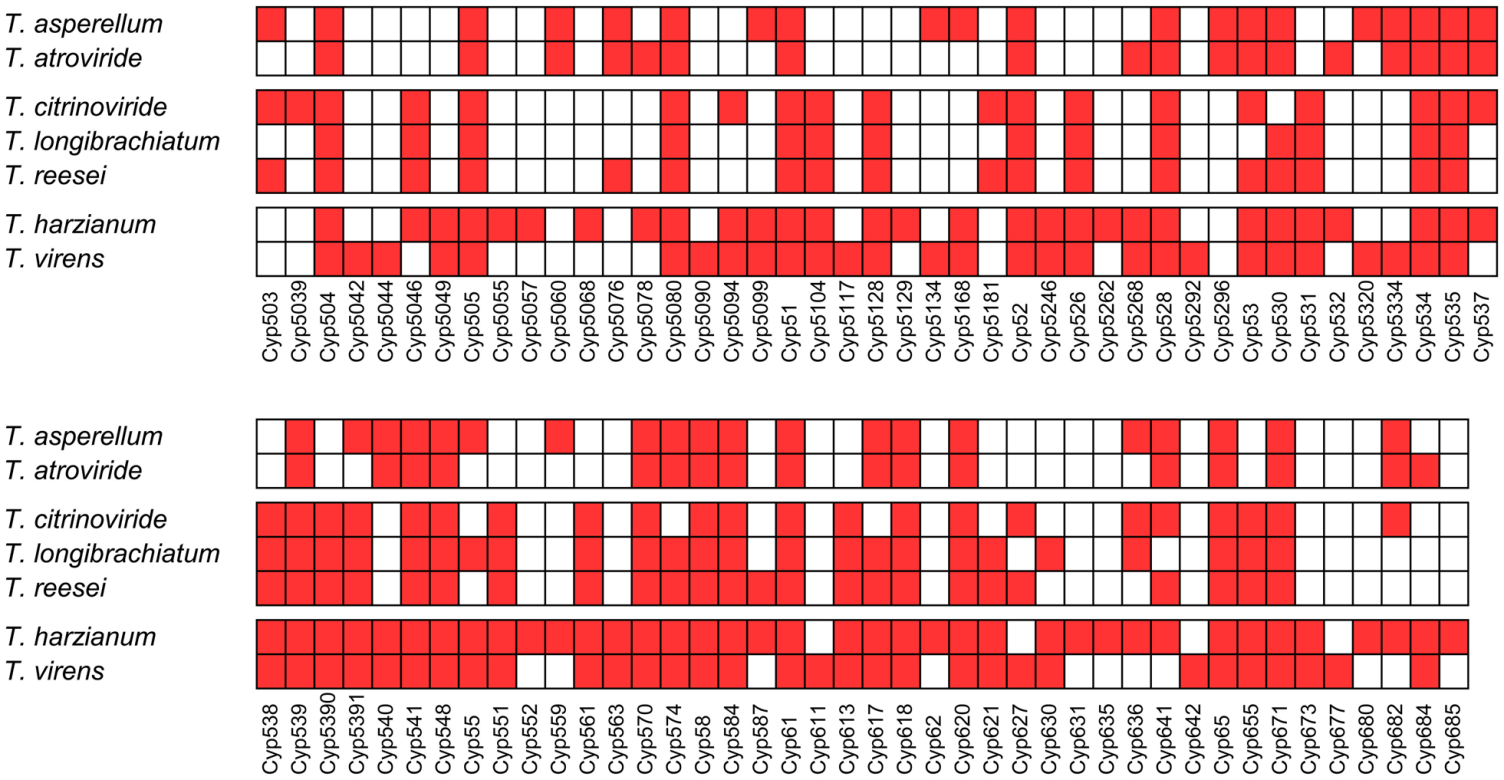
Cytochrome P450 families identified in *Trichoderma*

**Fig. 2:**
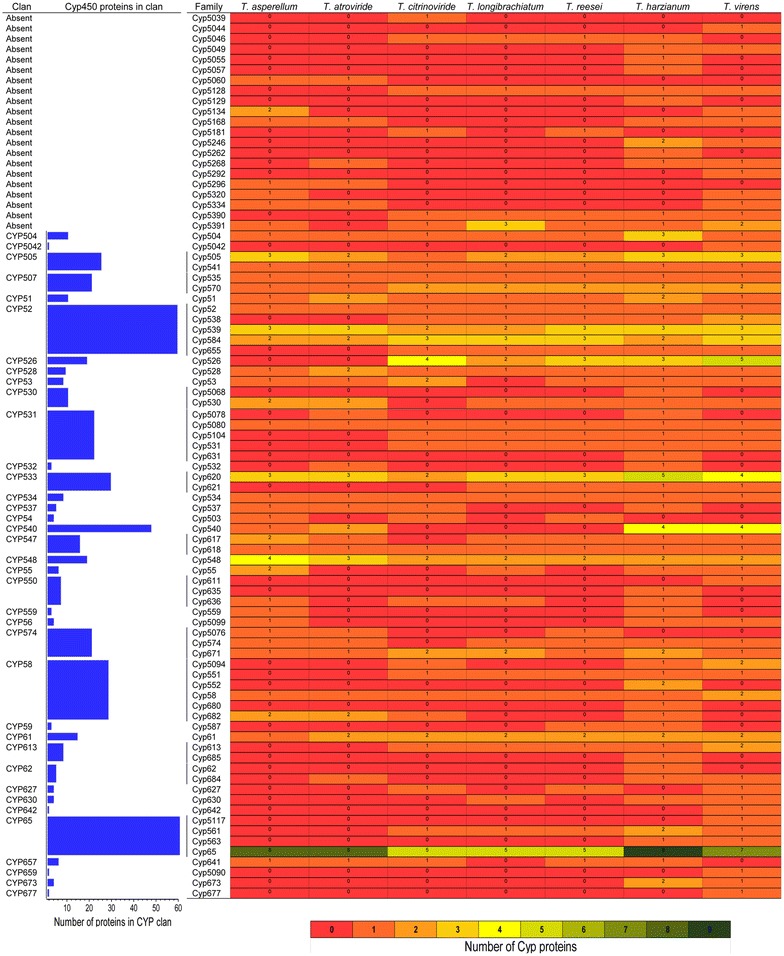
Abundance of cytochrome P450 families and clans in *Trichoderma*. The heatmap displays the abundance of Cyp protein families among *Trichoderma* species. Blue bar represent the number of Cyp proteins in a clan

**Fig. 3:**
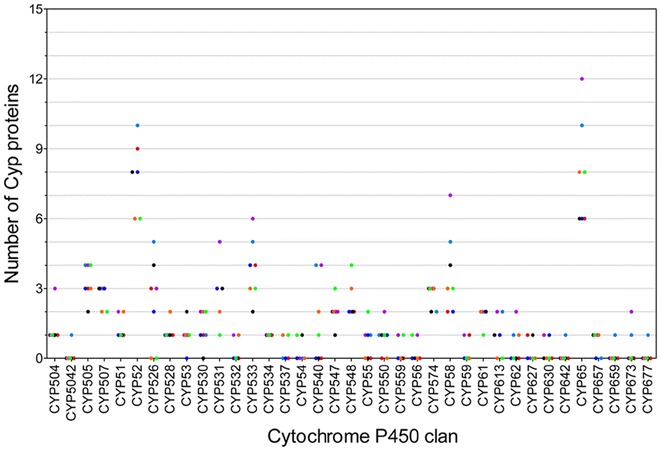
Diversity of cytochrome P450 clans among *Trichoderma* species. Scatter-plot presents number of proteins in CYP clans in *T. asperellum* (orange), *T. atroviride* (green)*, T. citrinoviride* (black), *T. harzianum* (purple), *T. longibrachiatum* (dark blue), *T. reesei* (red) and *T. virens* (light blue)

Identified cytochrome P450 proteins were annotated and classified into 85 families (Fig. 1) and 37 clans (Fig. 2). *Trichoderma* species showed diversity in the number of annotated Cyp families (Table 1, Figs. 1, 2, 3). The numbers of annotated Cyp families among *Trichoderma* species ranged from 36 (*T. atroviride*) to 67 (*T. harzianum*). Annotated CYP clans were also found to be diverse in *Trichoderma* (Fig. 3). The highest numbers of CYP clans were identified in *T. harzianum* (31) and *T. virens* (31). *T. asperellum* and *T. atroviride* contained 25 and 22 clan types respectively. Clans CYP52 and CYP65 were found to be most abundant with 55 and 56 protein entries, respectively (Fig. 3). The number of proteins in the most abundant clans CYP52 and CYP65 ranged from 6 to 12 among *Trichoderma* species. Clan CYP673 was identified only in *T. virens* and *T. harzianum,* containing 1 and 2 members respectively, and was found to be absent in other five species. Similarly, clan CYP56 proteins were found to be unique to *T. asperellum*, *T. harzianum* and *T. virens* with single entries in each species. Clan540 proteins were found absent in *T. citrinoviride*, *T. longibrachiatum* and *T. reesei*. Clans CYP5042, 642, 659 and 677 were identified only in *T. virens* and were absent in all other species.

### Phyletic distribution of CYP families and clans in *Trichoderma*

**Fig. 4:**
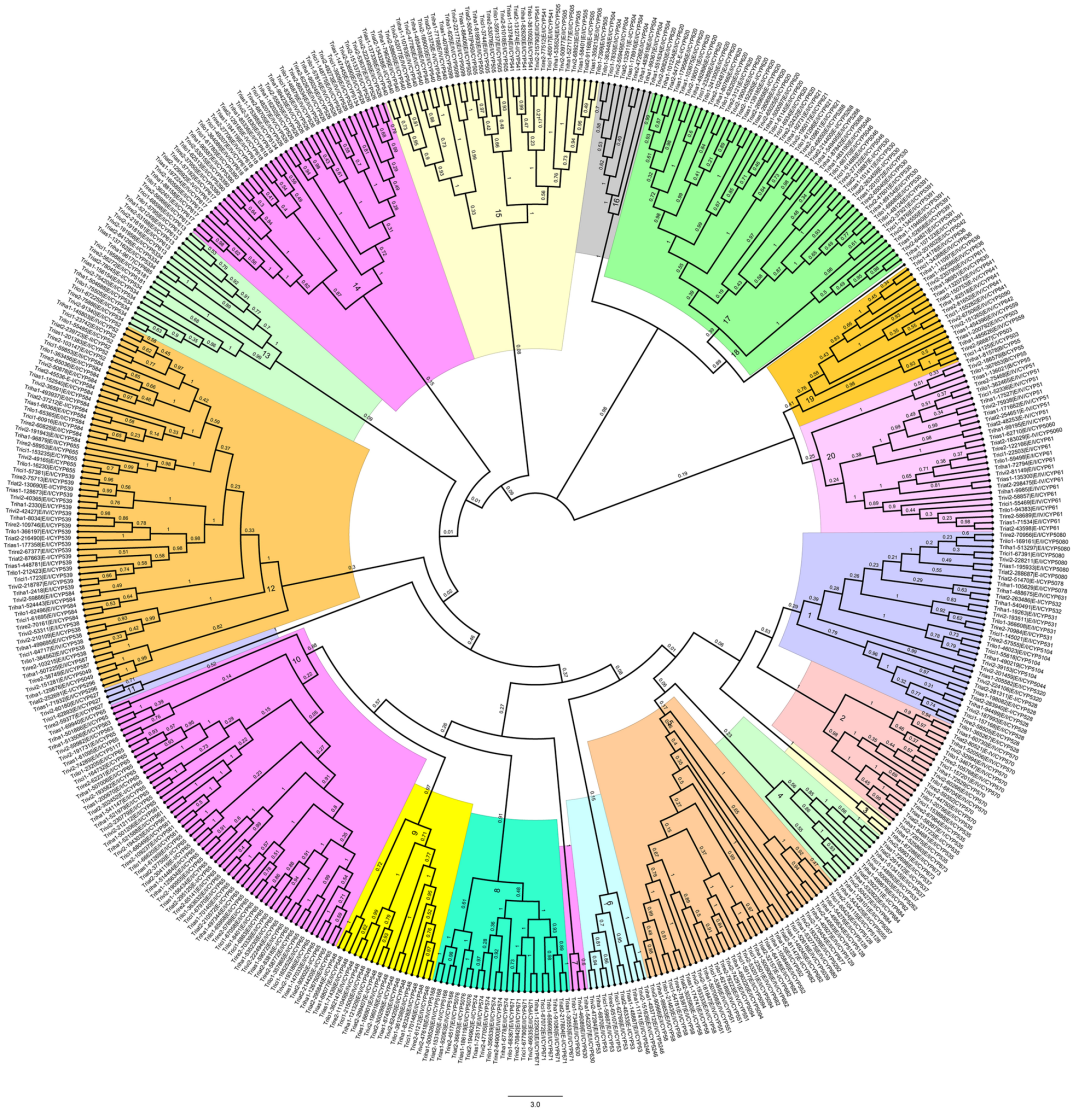
Evolutionary relationships of cytochrome P450 proteins among *Trichoderma* species. Phylogenetic tree was constructed inferred using the minimum evolution method^72^ using MEGA5 software. Phylogenetic groups (1–20) and bootstrap frequencies are shown in the tree. Tree includes Cyp proteins from all seven *Trichoderma species* including *T. asperellum*, *T. atroviride, T. citrinoviride*, *T. harzianum*, *T. longibrachiatum, T. reesei* and *T. virens*. Each phylogenetic group is indicated by a specific color

The genome-wide comparisons and annotations of P450s have allowed us to further develop the relationships among Cyp families in different *Trichoderma* species. To demonstrate the divergence of the primary sequences and evolutionary relationships of cytochrome P450 families in *Trichoderma*, a detailed phylogenetic analysis was carried out using 477 aligned Cyp protein sequences. The phylogenetic tree depicting evolutionary relationships among *Trichoderma* cytochrome P450 proteins are illustrated in Fig. 4. Further, the distribution of different CYP clans and families in 20 phylogenetic groups with their putative functions are summarized in Table 2.

Evolutionary studies differentiated 477 cytochrome P450 proteins from 7 *Trichoderma* species into 20 phylogenetic groups (Fig. 4). Group 1 consisted of a total of 33 Cyp proteins from clans CYP528, CYP531 and CYP532. In *Trichoderma*, clan CYP531 consists of five Cyp families including Cyp5078, Cyp5080, Cyp5104, Cyp531 and Cyp631. Group 2 consisted of total 19 protein members belonging to clan CYP507. Members of clan CYP507 have been predicted to be involved in xenobiotic metabolism in Pezizomycotina [36]. In FCPD, clan CYP507 consists of four Cyp families including Cyp 507, Cyp525, Cyp535 and Cyp570. Of these four families, only Cyp535 and Cyp570 families are present in *Trichoderma* species. Group 2 containing clan CYP507 proteins was further differentiated into two sub-groups containing families Cyp535 (7 proteins) and Cyp570 (12 proteins) respectively. In *Trichoderma*, all 19 proteins belonging to clan 507 are grouped together in group 2 suggesting conserved putative role of Cyp535 and Cyp570 in xenobiotics metabolism. Clan CYP673 in group 3 consists of only three members-two from *T. harzianum* and one from *T. virens.*

Group 4 consists of 11 proteins from 2 clans (CYP537 and CYP62). In FCPD, clan CYP537 consists of two families: Cyp537 and Cyp577. In *Trichoderma*, Cyp577 family is absent and Cyp537 proteins are present only in *T. asperellum, T. atroviride*, *T. citrinoviride* and *T. harzianum*. In group 4, all identified members of clan CYP62 grouped together. Clan CYP62 in FCPD consists of three CYP families including CYP62, CYP626 and CYP684. In *Trichoderma,* one Cyp62 (*T. harzianum*) and three Cyp684 proteins (one each) were identified in *T.* atroviride, *T. harzianum* and *T. virens*. Group 4 also contained Cyp50555, Cyp5057 and Cyp5262 proteins. The corresponding clans for these three families are absent in FCPD. Protein Cyp5262 was grouped together with members of clan CYP537, whereas Cyp50555 and Cyp5057 proteins formed a separate subgroup in Group 4. Group 5 contained total 35 proteins belonging to clans CYP58 and CYP677, which includes diverse Cyp families Cyp5039, Cyp5094, Cyp5128, Cyp5129, Cyp5292, Cyp551, Cyp552, Cyp58, Cyp677, Cyp680 and Cyp682. Clans for Cyp families 5039, 5128, 5129 and 5292 are not available in FCPD. *Trichoderma* has only one Cyp677 protein i.e., in *T. virens* which was grouped closely with Cyp5292 in phyletic Group 5. A total of 26 proteins belonging to clan CYP58 are identified in *Trichoderma*. Cyp58 family had a single member in all *Trichoderma* species analysed except in *T. virens* (2 proteins). All 7 members of CYP53 clan were grouped together in Group 6. Cyp53 family was found in all *Trichoderma* species except *T. longibrachiatum*. These proteins are involved in xenobiotic metabolism. The group 6 also contained three Cyp5246 proteins, clan for this family is absent in FCPD. Family Cyp5246 is present only in *T. harzianum* and *T. virens*. Members of Cyp53 and Cyp5246 families were differentiated in two clear sub-groups. Group 7 consists of only three proteins belonging to clan CYP630; one each from *T. harzianum*, *T. longibrachiatum* and *T. virens*. The group 8 consists of 23 proteins from clan CYP574 including families Cyp5076, Cyp574 and Cyp671. Four members of Cyp5168 family were also clustered in group 8.

Group 9 consists of all 17 proteins of clan CYP548. In *Trichoderma*, Cyp548 family is ubiquitously present in all seven species, where *T*. *asperellum* and *T. atroviride* contained four and three proteins respectively followed by two each in *T. citrinoviride*, *T. harzianum, T. longibrachiatum*, *T. reesei* and *T. virens.* These proteins are known to be involved in xenobiotic metabolism. The second largest phylogenetic Group 10 has 56 Cyps from the clan CYP65 which are involved in secondary metabolism. It comprised of families Cyp5117, Cyp561, Cyp563 and Cyp65. Group 11 consists of three Cyp627 proteins.

In *Trichoderma,* group 12 is the largest with 62 Cyp proteins. These Cyps from clans CYP52 and CYP59 were differentiated separately into two sub-groups. Clans CYP52 and CYP59 involve members of Cyp52, Cyp538, Cyp539, Cyp584, Cyp587 and Cyp655 families. Two entries of Cyp587 family belonging to clan CYP59 were grouped together with two proteins each from Cyp5049 and Cyp5296 families. The corresponding clan for Cyp5049 and Cyp5296 families were found to be absent in FCPD. In group 12, Cyp proteins of clan CYP52 were grouped together in the separate sub-group. Group 13 contained 19 Cyps belonging to clans CYP534 and CYP613. Two Cyp proteins belonging to family Cyp5181 were also present in group 13. Protein members of groups 12 and 13 were predicted to be involved in xenobiotic metabolism (Table 2).

Group 14 consists of proteins belonging to clans CYP526 and CYP547 which were differentiated separately into two sub-groups. In *Trichoderma*, 2 Cyp families of clan CYP547 were identified that includes Cyp617 (7) and Cyp618 (7). Cyp5134 proteins were grouped together in sub-group containing clan CYP526 proteins. Group 15 consists of 36 proteins involved in primary metabolism that includes members of clans CYP505 (15), CYP540 (11), CYP541 (7) and Cyp5099 (3). All three Cyp5099 proteins belonging to clan CYP56 family were included in this group. These proteins were identified only in *T. asperellum*, *T. harzianum* and *T. virens.* Cyp5099 proteins were found closely related to Cyp540 proteins and together formed a separate sub-group. Another sub-group contained all proteins belonging to clan CYP505 which includes Cyp505 and Cyp541 families. All nine protein members of clan CYP504 were clustered together in group 16. These proteins are known to be involved in xenobiotic metabolism. *Trichoderma* species contain single copy of Cyp504 protein except *T. harzianum* which contains three copies of Cyp504 protein involved in phenylacetate catabolism [37].

Group 17 is the third largest Cyp group consisting of 51 Cyps from clans CYP530 and CYP533. In this group, CYP533 is the most dominant clan followed by CYP530. Clans CYP530 and CYP533 include Cyp families Cyp530 (8 proteins) and Cyp5068 (1 protein), and Cyp620 (23 proteins) and Cyp621 (4 proteins) respectively. This group also contained Cyp5046 (4), Cyp5391 (8) and Cyp5268 (3) proteins. The corresponding clans for these families are absent in FCPD. Group 18 contains one Cyp5042 protein of *T. virens*. Group19 includes 18 proteins belonging to clans CYP54, CYP550, CYP559, CYP642, CYP657 and CYP659. These clans are involved in secondary metabolism. A total of 29 proteins from 7 *Trichoderma* species corresponding to three clans including CYP51, CYP55 and CYP61 were clustered together in group 20. These are known to be involved in primary metabolism. In this group, CYP51 and CYP61 families dominate with 9 and 15 members respectively. Further, all proteins belonging to Cyp51 were grouped together in group 20. This suggests that Cyp51 protein which is involved in primary metabolism (sterol biosynthesis) is diversified only to a lesser extent in *Trichoderma.* In comparison to some of the ascomycetous fungi, which carry multiple CYP51 proteins, *T. atroviride* and *T. harzianum* contained two copies each, whereas *T. asperellum, T. citrinoviride, T. longibrachiatum, T. reesei* and *T. virens* contained only single copy of Cyp51 protein.

### Characteristic motifs of the *Trichoderma* CYP families

**Fig. 5:**
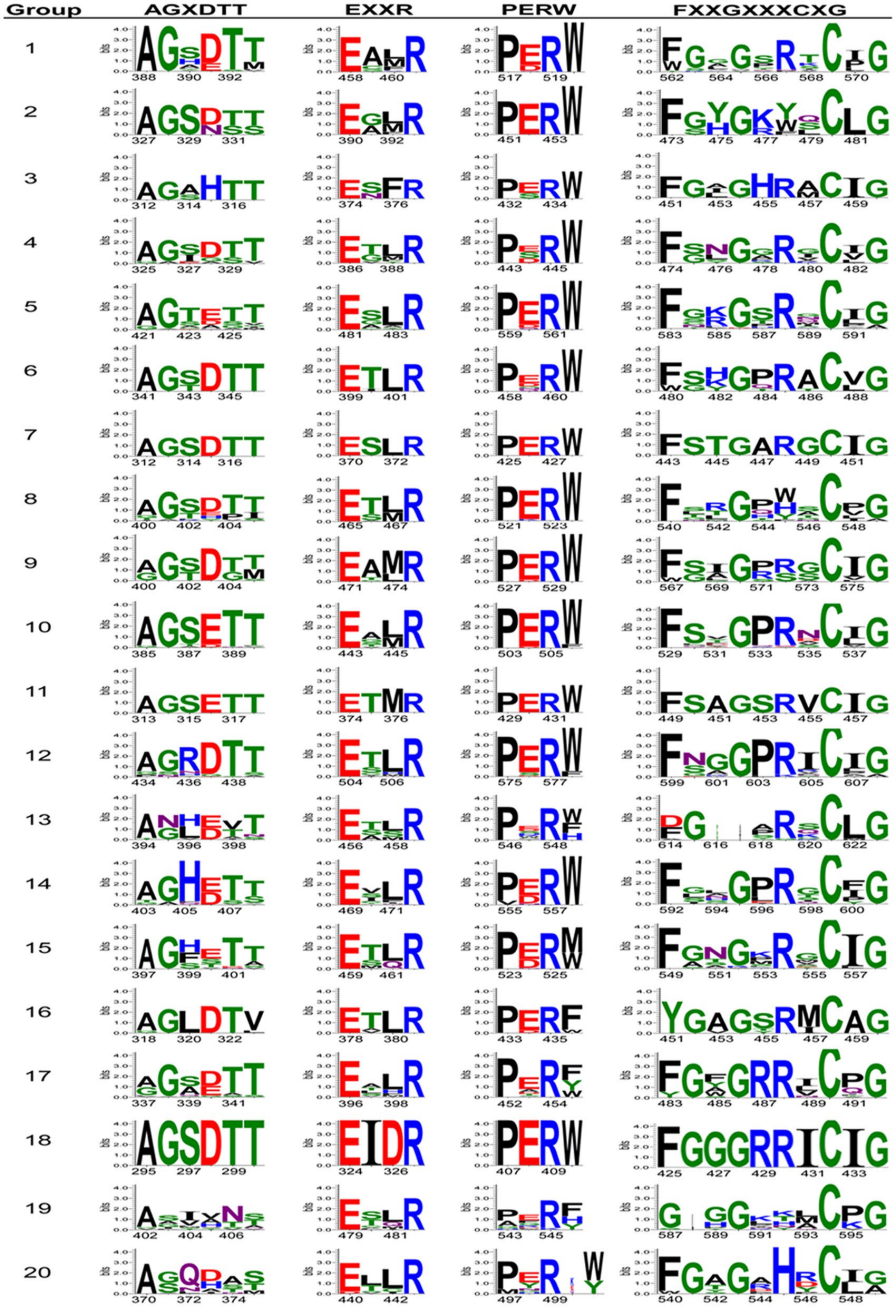
Conserved signature motifs of *Trichoderma* Cyp proteins for 20 phylogenetic groups

Several signature motifs are conserved in fungal Cyp proteins as per pervious findings [26, 35, 37, 38]. In *Trichoderma*, we identified the characteristic signature motifs of CYP super family AGXDTT, EXXR, PERW and FXXGXRXCXG for each phylogenetic group (Fig. 5). These motifs are functionally essential for the Cyp proteins. Conserved motif FXXGXRXCXG (also known as CXG) is designated as a heme-binding domain [26, 29, 39] and includes a conserved cysteine residue that binds to the Fe of the heme. In *Trichoderma*, the cysteine residue of the P450 signature CXG motif is invariantly conserved in all P450s, whereas two glycine and one phenylalanine residues were also found to be conserved among majority of phylogenetic groups, which are in accordance with previous reports [37, 40]. In phylogenetic groups 13, 16 and 19, Cyp proteins contain glutamate/aspartate, tyrosine and glycine respectively instead of a phenylalanine residue. Another variant of FXXGXRXCXG motif was found in groups 1, 6 and 20 where first amino acid residue of the motif was either phenylalanine or tryptophan. Further, in groups 5, 8, 12 and 20, FXXGXRXCXG and FXXGXRXCXA variants were identified. Conserved motif EXXR is present in helix K, on the proximal side of heme and probably is involved in the stabilization of the core structure of Cyp proteins [26, 35, 39]. In *Trichoderma* motif EXXR, glutamic acid and arginine residues were found to be highly conserved, whereas two middle ‘XX’ residues were found to be highly variable. These results are in concurrence with previously reported literature for fungal cytochrome P450 proteins [35, 37, 40, 41]. Another conserved motif of cytochrome P450 protein family is PERW (known as PER) which forms E-R-R triad in Cyp proteins [26]. In *Trichoderma*, we found PERW as the predominant signature, in accordance with previous reports in fungi [27, 35]. Motif PERW was found to be relatively conserved in *Trichoderma* with few exceptions that mainly includes phylogenetic groups 19 and 20. Group 19 consists of Cyp proteins from clans CYP54, CYP550, CYP559, CYP642, CYP657 and CYP659, which have been predicted to play role in secondary metabolism. High diversity of PER motif of this group could be attributed to the evolving functions of Cyp P450 protein members. Phylogenetic group 20 consisting of clans CYP51, CYP61 and CYP55 includes Cyp proteins belonging to both class E (CYP51 and CYP61) and B (CYP55). In this group, variant of PERW motif was identified where clan CYP55 proteins (class B) contained amino acid residues K/E/Q between PER and W/Y. The absence of an amino acid residue between arginine and tryptophan residues in “PERW” motif in all class E Cyp proteins indicate the early functional divergence of PERW motif in class B and E cytochrome P450 proteins. These results provide an insight on the structure–function relationships in such a diverse and complex Cyp protein families. Further, we also identified conserved motif, AGXDTT in *Trichoderma* cytochrome P450 proteins. Motif AGXDTT contributes to oxygen binding and activation [35]. The oxygen-binding domain (AGXDTT) was found to be highly variable in *Trichoderma* cytochrome P450 proteins. The terminal threonine residue in AGXDTT motif involved in the formation of the enzyme’s critical oxygen-binding pocket was found to be replaced predominately by valine in phylogenetic group 16. Other amino acid residues that replaced terminal threonine in different groups included serine or methionine. For motifs AGXDTT and CXG, Cyp proteins in phyletic groups 13, 19 and 20 (Table 2) were relatively less conserved, suggesting divergence of these Cyp protein sequences and their functions in *Trichoderma*. We found that the conserved signature motifs and their variants identified in *Trichoderma* showed few exceptions to previous reports. These results suggest Cyp signature motifs have evolved in *Trichoderma* to accommodate enormously wide range of substrate specificities and their substrate-binding regions.

### Cytochrome P450s associated with secondary metabolism related gene clusters

A survey of the genomes of seven *Trichoderma* spp. revealed that of the 477 cytochrome P450 genes present in the seven genomes, as many as 100 genes are associated with putative secondary metabolism related gene clusters namely NRPS, PKS, NRPS-PKS, NRPS-like, and terpene cyclase clusters (Additional file 1: Table S2).

## Discussion

*Trichoderma* species are the champions of opportunistic success [1]. They can be found virtually in all ecological niches, both terrestrial and aquatic. These fungi are capable of parasitizing a wide range of fungal and oomycetes species. Many species are known to colonize the rhizosphere and roots, both externally and internally [3]. Some are reported to be endophytes [42] while a few are aggressive parasites on cultivated mushrooms [43]. A few species are known to be opportunistic human pathogens while some strains are nematode-parasite, demonstrating their ability to parasitize members of animal kingdom [1]. Several *Trichoderma* strains are plant growth enhancers and some can colonize composts [44]. A few strains are known to be xenobiotics degraders. Most species are prolific producers of a wide range of secondary metabolites, with a total of more than 1000 compounds chemically characterized [45]. Cytochrome P450s are important for cells to perform a wide variety functions like primary and secondary metabolism, xenobiotic degradation and cellular defence (e.g., in interaction with other fungi). Recently, a *T. virens* P450 (TvCyt2; Protein Id. 190045) has been shown to be involved in biocontrol and plant growth promotion [46]. Basidiomycetes are capable of metabolizing a wide range of endogenous and exogenous compounds by using cytochrome P450s [47]. Great deal of information is available on the role of P450s in degradation of lignins and polyaromatic hydrocarbons by white rot fungus *Phanerochaete chrysosporium* and brown-rot fungus *Postia placenta*, as well as medicinal mushrooms like *Coriolus versicolor* and *Lentinula edodes* [48–51]. Role of P450s in colonization of living wood by the plant pathogen *Heterobasidion irregulare* is also well established [52].

In the present study, *Trichoderma* CYPome from seven *Trichoderma* species viz. *T. asperellum*, *T. atroviride*, *T. citrinoviride*, *T. harzianum*, *T. longibrachiatum*, *T. reesei* and *T. virens* is annotated. Overall, our analysis identified a total of 477 CYPs in these genomes. To provide support for the annotation process, the identified CYPs were also examined for conserved CYP domain. Our analysis of the CYPome has identified 12 families unique to *Trichoderma*. All the *Trichoderma* species examined are a rich source of Cyp proteins (55 in *T*. *longibrachiatum* to 100 in *T*. *harzianum*).

In *Trichoderma*, clan CYP52 consisted of families Cyp52, Cyp538, Cyp539, Cyp584 and Cyp655. Cyp52 family is found only amongst *Candida*-related species of fungi and these proteins catalyze the conversion of fatty acids and alkanes to alpha, omega-dicarboxylic acids [53]. The number of Cyp61 proteins was conserved in all *Trichoderma* species and these proteins were also grouped together in Group 20. Cyp61 proteins are involved in primary metabolism. In *Saccharomyces cerevisiae*, CYP61 codes for sterol 22 desaturase [54], which is involved in later stages of the ergosterol pathway in metabolizing Ergosta-5,7,24(28)-trienol to Ergosta-5,7,22,24(28)-tetraenol by introducing a C-22(23) double bond in the sterol side chain. Since Cyp61 is involved in the later stages of ergosterol pathway, it is considered to have evolved as a result of duplication and diversification of the CYP51 gene. In ascomycetes and basidiomycetes, clan CYP51 is involved in sterol biosynthesis and is recognized as the housekeeping CYP, and has been a popular antifungal target for the control of fungal diseases in humans and crop plants [29–31, 55]. In comparison to some of the ascomycetous fungi, which carry multiple CYP51 genes, *T. atroviride and T. harzianum* contained two copies whereas *T. asperellum, T. citrinoviride, T. longibrachiatum, T. reesei* and *T. virens* contained only single copy of Cyp51 protein. In addition, all members of both clans CYP51 and CYP61 which are involved in primary metabolism (sterol biosynthesis) are grouped together in group 20, suggesting that both Cyp51 and Cyp61 proteins are diversified only to a lesser extent in *Trichoderma.*

Motif analysis led to the identification of four signature motifs in phylogenetic groups, which correspond to the conserved tertiary structure and enzyme functions in spite of the wide sequence diversity and functions of Cyp proteins. Modifications found in the heme-binding domain FXXGXRXCXG are more frequently found in CYPs with catalytic activity, often not requiring oxygen [29]. These results indicate Cyp members of groups 5, 8, 12, 13, 16, 19 and 20 may have novel catalytic activities in *Trichoderma*. Some P450s showed variations of the signature motifs mainly in AGXDTT, EXXR and FXXGXRXCXG motifs. These results are in accordance with previous reports [38, 41] where it was proposed that these P450s variations may be due to misaligned sequences or that the P450s are missing the invariant residues at these motifs. In our study, phylogenetic group 10 containing protein families of clan CYP65 showed highly conserved motifs, suggesting functional conservation of CYP65 clan in analysed *Trichoderma* species. All members of clan CYP65 are involved in secondary metabolism. CYP65 is reported to catalyze the epoxidation reaction during the synthesis of trichothecenes [56, 57] and radicicol [37]. Identification of conserved and variable CYP motif signatures among and within phylogenetic groups in the present study may provide us information on CYP evolution, structure, and function in *Trichoderma* and have application in classification of proteins in gene expression analysis [58].

Cyp56 clan, found to be unique to *T. asperellum*, *T. harzianum* and *T. virens* (mycoparasites) has been characterized earlier in yeast [59, 60]. Members of Cyp56 clan are involved in meiotic spore wall biogenesis, particularly in dityrosine biosynthesis [59–61]. Members of the clan CYP507, CYP530, CYP531, CYP532 and CYP548 are known to be involved in xenobiotics metabolism [36]. Abundance of these proteins in *Trichoderma* may be related to the ability of these fungi to metabolize a wide range of xenobiotics, including many fungicides. Similarly, ability of *Trichoderma* spp. to produce a plethora of secondary metabolites could be linked to the abundance of P450s belonging to the clan CYP574, CYP58 and CYP65 proteins that have been implicated in trichothecene biosynthesis [62]. In *T. harzianum*, three copies of Cyp504 protein are present as compared to single copy in other *Trichoderma* species. Expansion of Cyp504 proteins in *T. harzianum* suggest important role of Cyp504 protein in xenobiotic metabolism. Further, the family members of Cyp504 were also reported to be up-regulated during cuticle infection by insect pathogenic fungi *Metarhizium anisopliae* and *M. acridum* [63]. Cyp505 family was found to be expanded in *T*. *asperellum*, *T. harzianum* and *T. virens* where these species contained three Cyp505 proteins each. Cyp505 proteins are membrane-associated fatty acid hydroxylase [64]. Cyp528 family has only one protein entry in all *Trichoderma* species analysed except *T. atroviride* where family Cyp528 consisted of two proteins. Similarly, Cyp58 family has a single protein entry in all *Trichoderma* species analysed except in *T. virens* where family Cyp58 consisted of two proteins. Previous studies also showed expansion of clan Cyp58 proteins in fungi [36]. In *Trichoderma*, the increase in CYPome size of *T. harzianum* and *T. virens* may be due to the expansion of certain *CYP* gene families or the presence of novel genes that are essential for their lifestyle. Previous reports have associated expansions of the fungal CYP families with the evolution of various fungal traits including pathogenicity [65]. Our phylogenetic analysis showed uneven distribution of CYP group sizes in *Trichoderma* species, which are in concordance with extreme expansions and contractions of certain CYP families in the course of evolution. Expansion of CYP clans in different *Trichoderma* species could aid them in more competent survival in their respective habitats.

*Trichoderma* spp. are prolific producers of secondary metabolites, many with antimicrobial, anticancer and plant growth-promoting properties [45] Cyps are known to play central role in biosynthesis if many, if not most of the secondary metabolites of plant and microbial origin. Till date, however, only a handful of *Trichoderma* Cyps have been investigated for their role in biosynthesis of secondary metabolites [46, 66, 67]. Our present findings suggest that more than 20% of the catalogued Cyps from *Trichoderma* are part of putative secondary metabolism-related gene clusters. There is a need for systematic studies on the functions of these Cyps which would lead to the discovery of novel pathways, metabolites and intermediates with greater biotechnological significance.

## Conclusion

*Trichoderma* CYPome described in our study is by combining information generated from existing databases, predicting conserved domains and identifying structural motifs in each hypothetical protein. By following internationally recognized nomenclature system, we have identified novel CYP clans and families unique to *Trichoderma*. Phylogenetic analysis elucidated distribution of Cyp families and clans in different evolutionary groups and their probable functions in metabolism or biosynthesis based on the comparisons with CYPomes of other organisms. The number of these proteins correlates with the genome size and many are species-specific. Unfortunately, the functions of none of these proteins are known. One reason being a lack of systematic studies and annotation of these proteins. Our CYPome annotation and evolutionary studies of seven *Trichoderma* species now provides opportunities for exploration of research-driven strategies to select *Trichoderma* species for various applications especially in relation to secondary metabolism and degradation of environmental pollutants. Several of these proteins could also have biotechnological applications like biotransformation and synthesis of pharmaceutically important drugs.

## Methods

### Sequence data

Sequences of Cytochrome P450s were retrieved from the Joint Genome Institute (JGI) fungal genome database MycoCosm (http://genome.jgi-psf.org/programs/fungi/index.jsf) for all the species of genus *Trichoderma*. The species included were *T. asperellum* (CBS 433.97) v1.0, *T. atroviride* (IMI 206040) v2.0, *T. citrinoviride* v4.0, *T. harzianum* (CBS 226.95) v1.0, *T. longibrachiatum* (ATCC 18648) v3.0, *T. reesei* (QM 6a) v2.0 and *T. virens* (Gv29-8) v2.0.

### Annotation of CYPs

The annotation pipeline of the CYPome in the *Trichoderma* species was done in a two-step procedure of identification and annotation. The identification step of CYP family was performed by using Conserved Domain Database (CDD); the cut-off of positive hits was set at *E*-value of 10^−2^. Entries with incomplete sequences and domain were manually removed from the data. Cyp proteins with complete conserved cytochrome P450 domains were further subjected to the annotation procedure using the Nelson’s P450 database against all named fungal cytochrome P450s (http://blast.uthsc.edu) with the *E*-value of 10^−4^ [68]. For annotation, sequence similarity cut-off of 40% was used. For few entries, we have followed criteria of the phenomenon called family creep that allows sequences less than 40% to be included in a family. For such entries, we have used sequence similarity cut-off of 30% and above. These predicted CYPs were then assigned to the corresponding family and clan types based on their highest homology according to the International P450 Nomenclature Committee Databases used by Nelson (http://drnelson.uthsc.edu/CytochromeP450.html) [21] and the fungal cytochrome P450 database (http://p450.riceblast.snu.ac.kr) [27] respectively.

### Structural feature analysis of CYP protein sequences

Presence of cytochrome P450 conserved domain was confirmed using conserved domain database [69]. To reveal phylogenetic group-specific conservation pattern of cytochrome P450 proteins, structural features were explored. To identify cyp conserved signature motifs, multiple protein sequence alignments for each phylogenetic group were built by MAFFT program [70] using E-INS-i iterative refinement method. Alignments were further refined and viewed using AliView [71]. Consensus logos of the alignments were automatically generated by WebLogo 3 program [72] and used for visualization of the conservation of signature motifs for each phylogenetic group. The generated logos were used for the analysis.

### Phylogenetic reconstruction of CYPs

After removal of redundant and incomplete sequences, the protein sequences were aligned using MUSCLE [73]. The evolutionary history was inferred using the minimum evolution method [74]. The bootstrap consensus tree inferred from 1000 replicates was taken to represent the evolutionary history of the taxa analysed [75]. The evolutionary distances were computed using the Poisson correction method [76] and are in the units of the number of amino acid substitutions per site. The rate variation among sites was modelled with a gamma distribution (shape parameter = 1). The ME tree was searched using the close-neighbor-interchange (CNI) algorithm [77] at a search level of 1. The neighbor-joining algorithm [78] was used to generate the initial tree. Evolutionary analyses were conducted in MEGA5 [79]. Phylogenetic trees were visualized with FigTree v1.1.2 [80].

### Identification of cytochrome P450s associated with secondary metabolism related gene clusters

A genome-wide survey was done to identify cytochrome P450s associated (presence in the vicinity) with secondary metabolism-related gene clusters, viz., NRPS, PKS, PKS/NRPS, NRPS-like and terpene cyclase clusters either manually (*T. reesei, T. virens and T. atroviride* [81, 82] or using automated pipeline on the respective genome pages (for *T. citrinoviride, T. longibrachiatum, T. asperellum* and *T. harzianum*).

## Additional file

**Additional file 1. Table S1:** List of Cyp protein entries with incomplete cytochrome P450 domain. **Table S2**: Cytochrome P450s associated with predicted secondary metabolism-related gene clusters.
