# Supplementary material for: Genome-wide analysis of cytochrome P450s of Trichoderma spp.: annotation and evolutionary relationships
Source: Fungal Biol Biotechnol. 2018 Jun 4;5:12. doi: 10.1186/s40694-018-0056-3 (PMC5985579; doi:10.1186/s40694-018-0056-3)
Supplement: Supplementary file 1 — Additional file 1. Table S1: List of Cyp protein entries with incomplete cytochrome P450 domain. Table S2: Cytochrome P450s associated with predicted secondary metabolism-related gene clusters. [file 40694_2018_56_MOESM1_ESM.pdf]

**Table S1.** List of Cyp protein entries with incomplete cytochrome P450 domain

| Trichoderma spp. | Incomplete entries | Incomplete terminus | Cytochrome P-450 domain (amino acid residues) | |
|---|---|---|---|---|
| | | | Start | End |
| *T. asperellum* | \|Trias1\|74589\| | C | 8 | 268 |
| | \|Trias1\|68713\| | N | 109 | 419 |
| | \|Trias1\|51693\| | N | 242 | 503 |
| | \|Trias1\|63192\| | N | 185 | 479 |
| | \|Trias1\|148485\| | N | 235 | 435 |
| | \|Trias1\|149507\| | N | 158 | 461 |
| | \|Trias1\|57351\| | N | 207 | 354 |
| | \|Trias1\|74377\| | N | 106 | 457 |
| | \|Trias1\|58239\| | N | 160 | 480 |
| | \|Trias1\|334617\| | N | 206 | 468 |
| | \|Trias1\|65994\| | N | 164 | 526 |
| | \|Trias1\|378536\| | Pseudogene | | |
| | \|Trias1\|53084\| | Pseudogene | | |
| | \|Trias1\|154835\| | Pseudogene | | |
| *T. atroviride* | \|Triat2\|313069\| | N | 188 | 432 |
| | \|Triat2\|156885\| | N | 242 | 503 |
| | \|Triat2\|174966\| | N | 52 | 349 |
| | \|Triat2\|293815\| | N | 227 | 483 |
| | \|Triat2\|296774\| | N | 141 | 456 |
| | \|Triat2\|303744\| | N | 135 | 518 |
| | \|Triat2\|38322\| | N | 319 | 488 |
| | \|Triat2\|293435\| | N | 183 | 564 |
| | \|Triat2\|216002\| | N | 169 | 509 |
| | \|Triat2\|79360\| | N | 208 | 328 |
| | \|Triat2\|293464\| | N | 938 | 994 |
| | \|Triat2\|88322\| | N | 959 | 1041 |
| | \|Triat2\|301918\| | Pseudogene | 101 | 139 |
| *T. citrinoviride* | \|Trici1\|9295\| | C | 104 | 390 |
| | \|Trici1\|7681\| | N | 219 | 414 |
| | \|Trici1\|2997\| | N | 5 | 85 |
| | \|Trici1\|54417\| | N | 198 | 461 |
| | \|Trici1\|59297\| | N | 166 | 454 |
| | \|Trici1\|58984\| | N | 241 | 495 |
| | \|Trici1\|66123\| | N | 174 | 506 |
| | \|Trici1\|66522\| | N | 226 | 538 |
| | \|Trici1\|148916\| | N | 216 | 528 |
| | \|Trici1\|149423\| | N | 139 | 248 |
| | \|Trici1\|61028\| | N | 331 | 517 |
| | \|Trici1\|59740\| | N | 36 | 381 |
| | \|Trici1\|168800\| | N | 1 | 375 |
| | \|Trici1\|170026\| | N | 322 | 466 |
| | \|Trici1\|54905\| | N | 318 | 537 |
| | \|Trici1\|64273\| | N | 113 | 418 |
| | \|Trici1\|2998\| | NC | 122 | 192 |

| | | | | |
|---|---|---|---|---|
| | \|Trici1\|169764\| | N | 24 | 318 |
| *T. harzianum* | \|Triha1\|47771\| | N | 2 | 169 |
| | \|Triha1\|525301\| | N | 210 | 518 |
| | \|Triha1\|554415\| | N | 245 | 435 |
| | \|Triha1\|94359\| | N | 141 | 493 |
| | \|Triha1\|100004\| | N | 100 | 453 |
| | \|Triha1\|498331\| | N | 208 | 493 |
| | \|Triha1\|5267\| | N | 234 | 429 |
| | \|Triha1\|481072\| | N | 246 | 519 |
| | \|Triha1\|21965\| | N | 53 | 486 |
| | \|Triha1\|86064\| | N | 104 | 419 |
| | \|Triha1\|93338\| | N | 49 | 483 |
| | \|Triha1\|114691\| | N | 234 | 505 |
| | \|Triha1\|552903\| | N | 143 | 495 |
| | \|Triha1\|7362\| | N | 55 | 524 |
| | \|Triha1\|549187\| | N | 21 | 311 |
| | \|Triha1\|99484\| | C | 44 | 236 |
| | \|Triha1\|145743\| | N | 653 | 1014 |
| *T. longibrachiatum* | \|Trilo1\|56127\| | N | 191 | 368 |
| | \|Trilo1\|60327\| | N | 191 | 479 |
| | \|Trilo1\|356685\| | N | 193 | 504 |
| | \|Trilo1\|367454\| | N | 75 | 183 |
| | \|Trilo1\|361148\| | N | 246 | 519 |
| | \|Trilo1\|3525\| | N | 322 | 525 |
| | \|Trilo1\|361541\| | N | 145 | 562 |
| | \|Trilo1\|58830\| | N | 279 | 456 |
| | \|Trilo1\|363943\| | N | 173 | 487 |
| | \|Trilo1\|45702\| | N | 351 | 545 |
| | \|Trilo1\|51748\| | N | 559 | 340 |
| | \|Trilo1\|60572\| | N | 221 | 511 |
| | \|Trilo1\|205473\| | N | 315 | 534 |
| | \|Trilo1\|4413\| | N | 2 | 75 |
| | \|Trilo1\|69955\| | C | 100 | 257 |
| *T. reesei* | \|Trire2\|54166\| | N | 216 | 393 |
| | \|Trire2\|4999\| | N | 165 | 445 |
| | \|Trire2\|65360\| | N | 159 | 478 |
| | \|Trire2\|73344\| | N | 322 | 517 |
| | \|Trire2\|66453\| | N | 234 | 493 |
| | \|Trire2\|82133\| | N | 229 | 520 |
| | \|Trire2\|69648\| | N | 113 | 456 |
| | \|Trire2\|64377\| | N | 142 | 415 |
| | \|Trire2\|37827\| | N | 298 | 446 |
| | \|Trire2\|69883\| | N | 58 | 390 |
| | \|Trire2\|10312\| | N | 714 | 1026 |
| | \|Trire2\|120889\| | N | 328 | 524 |
| *T. virens* | \|Trivi2\|53375\| | N | 164 | 554 |

| | | | |
|---|---|---|---|
| \|Trivi2\|147960\| | N | 185 | 517 |
| \|Trivi2\|191659\| | N | 1 | 184 |
| \|Trivi2\|53058\| | N | 153 | 568 |
| \|Trivi2\|52099\| | N | 216 | 393 |
| \|Trivi2\|13758\| | N | 302 | 444 |
| \|Trivi2\|49153\| | N | 207 | 527 |
| \|Trivi2\|53278\| | N | 233 | 520 |
| \|Trivi2\|53366\| | N | 226 | 507 |
| \|Trivi2\|60000\| | N | 290 | 486 |
| \|Trivi2\|91346\| | N | 165 | 490 |
| \|Trivi2\|126116\| | N | 146 | 466 |
| \|Trivi2\|147207\| | N | 158 | 431 |
| \|Trivi2\|193168\| | N | 59 | 343 |
| \|Trivi2\|214982\| | N | 208 | 493 |
| \|Trivi2\|216144\| | N | 202 | 455 |
| \|Trivi2\|38198\| | N | 180 | 503 |
| \|Trivi2\|29262\| | N | 5 | 85 |
| \|Trivi2\|32911\| | N | 92 | 419 |
| \|Trivi2\|34391\| | N | 233 | 487 |
| \|Trivi2\|192422\| | N | 191 | 453 |
| \|Trivi2\|59906\| | N | 177 | 284 |
| \|Trivi2\|223842\| | N | 79 | 163 |
| \|Trivi2\|60068\| | N | 29 | 421 |
| \|Trivi2\|37402\| | N | 340 | 561 |
| \|Trivi2\|59079\| | N | 85 | 415 |
| \|Trivi2\|70946\| | N | 383 | 494 |
| \|Trivi2\|134374\| | N | 278 | 465 |
| \|Trivi2\|155466\| | N | 223 | 459 |
| \|Trivi2\|196674\| | N | 158 | 491 |
| \|Trivi2\|205975\| | C | 46 | 222 |
| \|Trivi2\|200187\| | N | 329 | 483 |

**Table S2.** Cytochrome P450s associated with secondary metabolism–related gene clusters

| **Species** | **Gene Cluster** | **No. of CYPs per cluster** | **Protein ID of Cyps** |
|---|---|---|---|
| *T. virens* | NRPS/ Trivi2:70629 | 1 | Trivi2:186579\|B/Cyp55 |
| | NRPS/ Trivi2:62540 | 1 | Trivi2:212112\|E/I/Cyp65 |
| | NRPS/ Trivi2:70770 | 1 | Trivi2:191958\|E/I/Cyp584 |
| | NRPS/Trivi2:70742 | 1 | Trivi2:191958\|E/I/Cyp584 |
| | NRPS/Trivi2:78708 | 1 | Trivi2:216161\|E/I/Cyp613 |
| | NRPS/Trivi2: 48850 | 1 | Trivi2:49165\|E/Cyp655 |
| | NRPS/Trivi2: 53833 & 28610 | 1 | Trivi2:60180\|E/I/Cyp627 |
| | PKS/Trivi2:47407 | 1 | Trivi2:47601\|E/I/Cyp530 |
| | PKS/Trivi2:51044 | 2 | Trivi2:50878\|E/I/Cyp584 |
| | NRPS-PKS/Trivi2:48850 | 1 | Trivi2:49165\|E/Cyp655 |
| | NRPS-PKS/Trivi2:53833, 28610 | 1 | Trivi2:60180\|E/I/Cyp627 |
| | NRPS-PKS/Trivi2:28610 | 1 | Trivi2-60180\|E/I/Cyp627 |
| | NRPS-PKS/Trivi2:78708 | 1 | Trivi2-216161\|E/I/Cyp613 |
| | NRPS-Like/Trivi2:60181 | 1 | Trivi2:60180\|E/I/Cyp627 |
| | TC/Trivi2:224958 | 2 | Trivi2:193582\|E/I/Cyp65<br>Trivi2:181847\|E/IV/Cyp551 |
| | TC/Trivi2:53145 | 1 | Trivi2:53201\|E/I/Cyp5094 |
| | TC/Trivi2:56195 | 3 | Trivi2:74291\|E/I/Cyp5094<br>Trivi2:74289\|E/I/Cyp5117<br>Trivi2:39153\|Cyp5104 |
| *T. atroviride* | NRPS/Triat2:39904 or 40716<br>NRPS/Triat2:10385 or 181616 | 1 | Triat2:152285\|E/I/Cyp620 |
| | PKS/Triat2:51726 or 188840 | 1 | Triat2:304119\|E/I/Cyp65 |
| | PKS/Triat2:45854 or 217234 | 1 | Triat2:45536-E/I/Cyp584 |
| | PKS/Triat2:45973 | 1 | Triat2:263459\|E/I/Cyp530 |
| | PKS/Triat2:32458<br>PKS/Triat:245485/267549 | 3 | Triat2:295844\|E/I/Cyp548<br>Triat2:84128\|E/I/Cyp5082<br>Triat2-295125\|E-I/Cyp65 |
| | NRPS-PKS/ Triat2:51715(212102) | 2 | Triat2:304119\|E/I/Cyp65,<br>Triat2:51470\|E-I/Cyp5043 |
| | NRPS-Like/Triat2:224197 | 1 | Triat2:319887\|E/I/Cyp530 |
| *T. reesei* | NRPS/Trire2:24586<br>NRPS/Trire2: 60751 | 1<br>1 | Trire2:53168\|E/I/Cyp613,<br>Trire2-27722\|E/I/Cyp5046 |
| | NRPS/Trire2: 271005 | 2 | Trire2: 38749\|E/I/Cyp587,<br>Trire2: 70984\|E/I/Cyp531 |
| | PKS/Trire2:65172 | 1 | Trire2:65036\|E/I/Cyp584 |
| | PKS/Trire2:65116 | 1 | Trire2:65040\|E/I/Cyp530 |
| | PKS/Trire2:82208<br>PKS/Trire2:73618 | 3 | Trire2:70161\|E/I/Cyp584<br>Trire2:37950\|E/I/Cyp617,<br>Trire2-102487\|E/I/Cyp620 |
| | NRPS-PKS/Trire2:58285 | 1 | Trire2:58953\|E/I/Cyp655 |
| | NRPS-PKS/Trire2:59315<br>NRPS-PKS/Trire2:4117 | 2 | Trire2:59377\|E/I/Cyp627<br>Trire2-78387\|E/I/Cyp58 |
| | NRPS/Trilo1: 1392769 | 1 | Trilo1: 5795\|E/I/Cyp613 |

| | | | |
|---|---|---|---|
| | NRPS/Trilo1: 165613 | 1 | Trilo1: 366608\|E/I/Cyp531 |
| | PKS/Trilo1:1470852 | 1 | Trilo1:363456\|E/I/Cyp584 |
| | PKS/Trilo1:1431707 | 1 | Trilo1:66680\|E/I/Cyp530 |
| | PKS/Trilo1:1434581 | 1 | Trilo1:62496\|E/I/Cyp584 |
| | PKS/Trilo1:1431707 | 2 | Trilo1:62513\|E/I/Cyp547,<br>Trilo1:241300\|E/I/Cyp620 |
| | NRPS-PKS/Trilo1:1459985 | 1 | Trilo1:16230\|E/Cyp655 |
| | NRPS-PKS/Trilo1:1471953 | 1 | Trilo1:21463\|E/I/Cyp58 |
| | TC/Trilo1:1406488 or 36731 | 2 | Trilo1:23205\|E/I/Cyp65<br>Trilo1:62189\|E/IV/Cyp551 |
| | TC/Trilo1:1442452 or 61665 | 1 | Trilo1:68704\|E/IV/Cyp570 |
| | NRPS/Trici1:1122832 | 1 | Trici1:21500\|E/IV/Cyp548 |
| | NRPS/Trici1:1129911 | 1 | Trici1:61488\|E/I/Cyp5046 |
| | NRPS/Trici1:1129911 | 1 | Trici1:68598\|E/I/Cyp613 |
| | PKS/Trici1:65461 | 1 | Trici1:52618\|E/IV/Cyp5039 |
| | PKS/Trici1:7685 | 1 | Trici1:59853\|E/II/Cyp584 |
| | PKS/Trici1:1186452 | 2 | Trici1:61780\|E/I/Cyp617<br>Trici1:61695\|E/I/Cyp584 |
| | NRPS-PKS/Trici1:1107986 | 1 | Trici1:62893\|E/I/Cyp627 |
| | NRPS-PKS/Trici1:1131145 | 1 | Trici1:153235\|E/Cyp655 |
| | TC/Trici1:5384 | 2 | Trici1:5385\|E/IV/Cyp551<br>Trici1:164732\|E/Cyp65 |
| | TC/Trici1:52419 | 2 | Trici1:4125\|E/I/Cyp503<br>Trici1:19588\|E/I/Cyp5181 |
| | NRPS/Triha1:15930 | 1 | Triha1:507225\|E/I/Cyp587 |
| | NRPS/Triha1:551513 | 1 | Triha1:81578\|B/Cyp55 |
| | NRPS/Triha1:80219 | 1 | Triha1:493712\|E/I/Cyp5246 |
| | NRPS/Triha1:496332 | 2 | Triha1:521249\|E/I/Cyp613<br>Triha1:483190\|E/I/Cyp5046 |
| | NRPS/Triha1:501208 | 1 | Triha1:501206\|E/I/Cyp561 |
| | NRPS/Triha1:7629 | 1 | Triha1-532775\|E/IV/Cyp680 |
| | PKS/Triha1:536358 | 3 | Triha1:40927\|E/IV/Cyp5129<br>Triha1:488675\|E/IV/Cyp631<br>Triha1:5\|E/I/Cyp552 |
| | PKS/Triha1:85730 | 2 | Triha1:495469\|E/I/Cyp540<br>Triha1:520506\|E/IV/Cyp570 |
| | PKS/Triha1:120155 | 1 | Triha1:521979\|E/I/Cyp65, 7362 |
| | PKS/Triha1:509408 | 1 | Triha1:145803\|E/IV/Cyp52 |
| | PKS/Triha1: 526013 | 1 | Triha1:501866\|E/I/Cyp65 |
| | NRPS-PKS/Triha1:488711 | 1 | Triha1:514484\|E/I/Cyp65 |
| | NRPS-PKS/Triha1:87171 | 1 | Triha1:87842\|E/I/Cyp5068 |
| | NRPS-PKS/Triha1:96798 | 2 | Triha1:9617\|Cyp685,<br>Triha1:96879\|E/II/Cyp655 |
| | TC/Triha1:113113 | 1 | Triha1:507006\|E/I/Cyp65 |
| | TC/Triha1:523651 | 1 | Triha1:507008\|E/IV/Cyp551 |
| *T. asperellum* | NRPS/ Trias1:75656 | 1 | Trias1:454396\|E/IV/Cyp559 |
| | NRPS/Trias1:74656 | 1 | Trias1:166901\|E/IV/Cyp548 |
| | NRPS/Trias1:79628 | 1 | Trias1:139185\|E/I/Cyp620 |
| | NRPS/Trias1:89466 | 1 | Trias1:147445\|E/I/Cyp5134 |

| | | | |
|---|---|---|---|
| | NRPS/Trias1:71435 | 1 | Trias1:71439\|E/I/Cyp548 |
| | PKS/Trias1:456732 | 1 | Trias1:137162\|E/I/Cyp5082 |
